# TESTING THE ASSOCIATION BETWEEN PSYCHOTROPICS AND OPIOIDS WITH FUNCTION IN HOSPITALIZED OLDER ADULTS WITH DEMENTIA

**DOI:** 10.1093/geroni/igae098.2365

**Published:** 2024-12-31

**Authors:** Nayeon Kim, Barbara Resnick

**Affiliations:** University of Maryland Baltimore, Baltimore, Maryland, United States; University of Maryland Baltimore, Baltimore, Maryland, United States

## Abstract

The aim of this study was to test if the use of psychotropic medications (antipsychotics, sedatives, antiseizure, antidepressants, and anxiolytics) and opioids were associated with physical function in hospitalized older adults living with dementia. This study was a secondary data analysis using baseline data from the Function Focused Care for Acute Care Using the Evidence Integration Triangle (FFC-AC-EIT) study, which is an ongoing randomized controlled trial. The first 369 participants were included. It was hypothesized that controlling for age, gender, race, comorbidities, cognitive function, and pain, the use of psychotropic medications and opioids would be negatively associated with physical function in the first 48 hours of admission. A path analysis was done to test this model using the AMOS statistical program. Overall, 72.5% (n=230) of the sample was on at least one psychotropic medication, and 18.8% (n=69) of the sample was on at least one opioid. Neither being on an opioid nor psychotropic medications was associated with function. Comorbidities, cognition, and pain were, however, significantly associated with physical function, such that those who had more comorbidities, better cognition, and less pain were more functionally independent. The findings support prior research showing a lack of association between psychotropic medication use and opioid use with physical function for hospitalized older adults. These drugs may be used appropriately to manage symptoms that allow for functional engagement of patients. Additional research is needed to continue to investigate the association of these medications with function and potential benefit versus harm.

